# Body Mass Index and Calprotectin Blood Level Correlation in Healthy Children: An Individual Patient Data Meta-Analysis

**DOI:** 10.3390/jcm9030857

**Published:** 2020-03-20

**Authors:** Anais Grand, Emmanuelle Rochette, Frederic Dutheil, David Gozal, Valeria Calcaterra, Roberto Berni Canani, Nazan Cobanoglu, Joep P. M. Derikx, Gianluca Terrin, Bruno Pereira, Etienne Merlin

**Affiliations:** 1 Department of Pediatrics, Clermont Ferrand University Hospital, F-63000 Clermont-Ferrand, France; anais.grand2@etu.uca.fr (A.G.); e_merlin@chu-clermontferrand.fr (E.M.); 2 Clermont Auvergne University, INSERM, CIC 1405, CRECHE unit, F-63000 Clermont-Ferrand, France; 3 Université Clermont Auvergne, CNRS, LaPSCo, Physiological and Psychosocial Stress, University Hospital of Clermont-Ferrand, CHU Clermont-Ferrand, Preventive and Occupational Medicine, WittyFit, F-63000 Clermont-Ferrand, France; fdutheil@chu-clermontferrand.fr; 4 Department of Child Health and Child Health Research Institute, University of Missouri School of Medicine, Columbia, MO 65201, USA; gozald@health.missouri.edu; 5 Department of Internal Medicine, Pediatric and Adolescent Unit, University of Pavia and Department of the Mother and Child Health, Pediatric Endocrinology Unit, Fondazione IRCCS Policlinico San Matteo, 27100 Pavia, Italy; v.calcaterra@smatteo.pv.it; 6 Department of Translational Medical Science and ImmunoNutritionLab at CEINGE Advanced Biotechnologies University “Federico II”, 80131 Naples, Italy; berni@unina.it; 7 Department of Pediatric Pulmonology, Ankara University, Faculty of Medicine, 06590 Ankara, Turkey; ncobanoglu@ankara.edu.tr; 8 Department of Pediatric Surgery, Emma Children’s Hospital, Amsterdam UMC, University of Amsterdam and Vrije Universiteit Amsterdam, 1100 DD Amsterdam, The Netherlands; j.derikx@maastrichtuniversity.nl; 9 Department of Mother and Child Health, University La Sapienza, 00161 Rome, Italy; gianluca.terrin@uniroma1.it; 10 Department of Biostatistics, Clermont Ferrand University Hospital, F-63000 Clermont-Ferrand, France; bpereira@chu-clermontferrand.fr; 11 Clermont Auvergne University, INRA, UMR 1019 UNH, ECREIN, F-63000 Clermont-Ferrand, France

**Keywords:** pediatric, S100A8/A9, weight

## Abstract

Background: Calprotectin (CP) is a protein complex involved in many inflammatory diseases. Obesity is characterized by low-grade inflammation and elevated circulating levels of calprotectin. However, associations between body mass index (BMI) and calprotectin levels have not been explored in otherwise healthy children. Methods: In accordance with Preferred Reporting Items for Systematic Reviews and Meta-Analyses (PRISMA) guidelines, we searched PubMed and Cochrane Library database up to July 2019. Healthy children’s blood calprotectin values were extracted, and potential correlations were explored. Results: A total of six studies that included data on 593 healthy children were identified. Median calprotectin value was 900.0 (482.0; 1700) ng·mL^−1^. Multivariable analysis showed no significant associations with age, sample type (serum vs. plasma), or sex. In contrast, a significant effect of BMI z-score (*p* < 0.001) emerged. Indeed, a positive correlation between BMI z-score and CP, was detected in girls (R: 0.48; *p* < 0.001) and boys (R: 0.39; *p* < 0.001). Conclusion: Calprotectin blood levels correlate with the degree of adiposity in healthy children, but are not affected by age, sex, or sample type (serum or plasma).

## 1. Introduction

Calprotectin (CP) is a protein complex composed of two subunits, S100A8 and S100A9, and primarily detected in polymorphonuclear neutrophils (PMN) [1]. When neutrophils are activated, CP is released and serves as an endogenous ligand to the toll-like receptor 4 (TLR4) on the surface on PMN. CP and TLR4 interactions lead to activation of a transcriptional signaling pathway that results in the up-regulation of pro-inflammatory cytokines such as tumor necrosis factor alpha (TNF-α) or interleukin 6 (IL-6). Thus, CP’s role as ligand of TLR4 induces the initiation and maintenance of an inflammatory state [2]. CP–TLR4 complexes are present in some body compartments, including blood, feces, and synovial fluid and have been implicated in several pediatric diseases [3]. Hence, CP’s role in inflammatory processes has led to its adoption as a reliable marker of subclinical systemic inflammation [4].

Overweight and obesity are generally associated with a low-grade inflammatory state, as evidenced by increased release of several proinflammatory cytokines and chemokines by adipose tissue. In adults, CP blood levels are higher in obese subjects and decrease proportionately to weight loss after Roux-en-Y gastric bypass surgery [5]. Similarly, CP blood levels are higher in obese children when compared to non-obese children [6], but the correlation between body-mass index (BMI) and CP blood levels has not specifically been addressed. The aim of this individual patient data meta-analysis was to determine correlation between blood level of CP and BMI z-score in otherwise healthy children.

## 2. Materials and Methods

### 2.1. Data Source

The present systematic review and individual patient data meta-analysis were carried out in accordance with the PRISMA statement (Preferred Reporting Items for Systematic Reviews and Meta-Analyses) [7]. Systematic search was limited to English articles with full text available on PubMed, Embase, Cochrane, and PsycINFO database from 1980 to July 2019.

### 2.2. Eligibility Criteria

The primary outcome was to determine correlation between blood levels of CP and BMI in healthy children. Secondary outcomes were potential associations between blood levels of CP and age, sex, and sample type (plasma or serum).

Studies included were case-control studies in which blood levels of CP were measured in healthy and sick children, or in healthy children and adults compared to sick children and adults. Our work exclusively focused on the data for healthy children. Studies were excluded if they included either only sick children, only adults, if only fecal levels of CP were measured, or if no healthy children were included in the study.

### 2.3. Search Strategy and Selection Criteria

Systematic search using title, abstract, and medical subject headings terms in order to identify relevant publications containing not less than one term from each of the three following categories of search terms used: “calprotectin”, “child”, and “plasma” or “serum”. The PubMed strategy is described in the Supplementary Materials.

We made an additional search for studies in accordance with the inclusion criteria through hand searching and previous review reference lists. This later search process was updated until September 2019.

### 2.4. Data Extraction, Evaluation, and Synthesis

**Figure 1:**
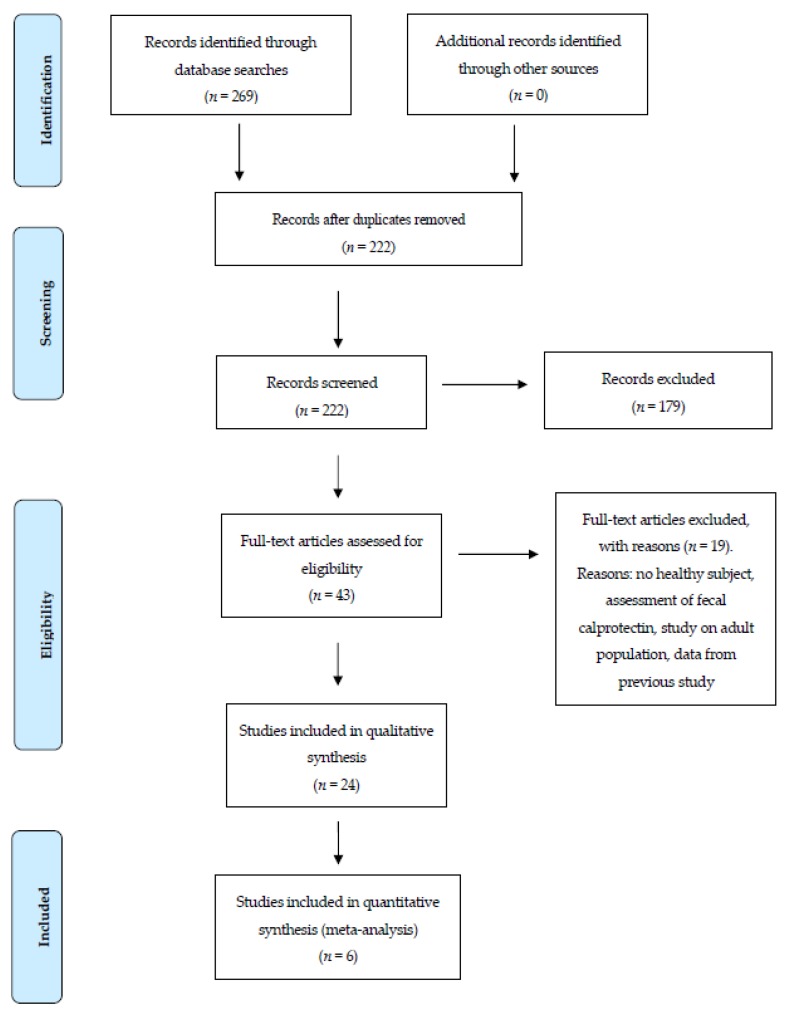
Studies selection following a Preferred Reporting Items for Systematic Reviews and Meta-Analyses PRISMA (Preferred Reporting Items for Systematic Reviews and Meta-Analyses) flow diagram. Studies were excluded when any healthy child was included, when fecal calprotectin was assessed and when normal values of blood calprotectin originated from another study.

Study selection followed PRISMA flow diagram (Figure 1). Relevant studies were reviewed in full text, such as to determine their relevance for the meta-analysis. After reading titles and eliminating duplicates (A.G. and E.R.), 43 abstracts were independently assessed by two authors (A.G. and E.R.), and from these, 5 references were subjected to detailed analysis and included in the sample by consensus or majority decision. In the case of conflictive classification of a study by the first two authors, another author (E.M.) examined the publication and a consensus was reached. Previous literature reviews were immediately excluded.

### 2.5. Data Extraction

We contacted all authors of the retained studies to obtain the individual datasets. Data of interest included: age (years), gender (1 = boy, 2 = girl), weight (kg), height (cm), BMI (if available), Tanner stage (if available), and CP values (ng·mL^−1^).

Data relating to general study information (authors, year of publication, sample size), study design (cross-sectional or cohort studies), participant characteristics (age, sex), plasma or serum sample, and results (mean and SD of calprotectin levels) were assembled in tabular format (Table 1).

### 2.6. Risk of Bias and Assessment of Study Quality

Methodological quality and risk of bias of included studies was determined by the Newcastle–Ottawa Scale (NOS) [8], which contains eight categories relating to methodological quality. Each study was given an eventual score out of a maximum of 10 points. A score of 0–4 points equated to a low quality study, a score of 5–6 points equated to a moderate quality study, a score of 7–8 points equated to a good study, and a score of 9–10 points was required for a study to be given a score of high quality (Figure S1). Eligible studies were independently rated by two authors blind to the study authors and institutions (A.G. and E.R.).

### 2.7. Statistical Considerations

The statistical analyses were performed using Stata software (version 13, StataCorp, College Station, TX, USA). For descriptive analyses, categorical data were presented with numbers and percentages, whereas continuous data were expressed as mean and standard-deviation or median and interquartile range, according to the statistical distribution. The assumption of normality was assessed by using the Shapiro–Wilk test. All analyses took into account between- and within-study variability. More precisely, random-effects models were preferred over the usual statistical tests to address the non-independence of data due to study effect. Thus, linear mixed models were used to study the variables associated to CP, i.e., age, sex, BMI z-score, and sample type. The normality of residuals and of random effects was determined using the Shapiro–Wilk test, and the logarithmic transformation of CP values was proposed to achieve the normality of the dependent endpoint (calprotectin). The intraclass correlation coefficient (ICC) related to the study effect was estimated. The relationship between continuous variables was studied using correlation coefficient (Pearson or Spearman, according to the statistical distribution). As CP values from Cobanoglu et al.’s study are two orders of magnitude lower than the other studies, a sensitivity analysis was performed excluding these data.

## 3. Results

### 3.1. Studies Selection

The schematic reflecting study selection is shown in Figure 1. Database searching led to identification of 269 articles. After duplicate removal, 222 articles were retained. Articles were then assessed based on title and abstract, which led to the elimination of 179 articles. Full text evaluation of 43 articles was then conducted with elimination of 19 articles. We contacted authors from each of the potentially viable articles and solicited the de-identified datasets corresponding to healthy children measurements included in their studies. Five authors agreed to collaborate while the others disagreed. Reasons for disagreement were as follows: data were not available for three studies (two were done in 1996 and one in 1997), one study was excluded because no healthy child actually took part in the study, and one was excluded because of author refusal. Thus, six studies were finally included in the individual patient data meta-analysis. The mean score of the quality of studies (NOS checklist) was 7.5 ± 0.7/10. The quality of studies selected for the meta-analysis was considered good.

We added 26 samples from our center, which were obtained in healthy children in the fasting state. Samples were immediately centrifuged, after collection, at 3000 *g* for 10 min at 4 °C and stored at −80 °C until analysis. Plasma CP levels were measured using a commercially-available ELISA assay (Bühlmann MRP8/14 Calprotectin ELISA kit; Bühlmann Laboratories, Schönenbuch, Switzerland), which has an inter-assay coefficient of variability (CV) of 5.8%, an intra-assay CV of 4.3%, and a low detection limit of 400 ng·mL^−1^. Analyses were performed in duplicate for all samples.

### 3.2. Study Characteristics

The characteristics of the six included studies are summarized in Table 1. Of the studies included in this meta-analysis, healthy subjects were compared to children with obstructive sleep apnea in two studies [9,10] and to children with either sepsis [11], suspected appendicitis [13], second-hand smoke [12], or obese children in one study each [6]. One of the authors [9,10] provided the data corresponding to two studies but also including unpublished results without further specification. These results are presented in a single line in Table 2 and Table 3.

### 3.3. Subject Characteristics

Subject characteristics are shown in Table 2. We included in the meta-analysis 567 healthy children from six studies [6,9,10,11,12,13] and 26 healthy children from unpublished data from our center. Among the 593 healthy children, there were 270 girls and 323 boys, with a mean age of 6.8 ± 4.4 years at the time of CP assay. CP was measured in plasma for three studies (*n* = 282) and in serum for the other three (*n* = 312). BMI was available for four of the studies, and the mean BMI z-score was 2.45 ± 2.3.

### 3.4. Meta-Analysis and Sensitivity Analysis

For all studies (*n* = 593), the median CP value was 900.0 (482.0; 1700.0) ng·mL^−1^, with an intraclass correlation coefficient (ICC) of 36% (Table 3). There were four studies that provided data of BMI z-score (Kim et al. [9,10], Cobanoglu et al. [12], Calcaterra et al. [6], and Merlin et al.); the pooled sensitivity value (*n* = 439) for median CP was 971.0 (431.0; 1840.0) ng·mL^−1^, with an ICC at 37%. A sensitivity analysis was carried out excluding Cobanoglu et al. [12] data. Multivariate analysis (*n* = 388) by study, age, sex, and BMI z-score showed no effect for age (*p* = 0.57) and sex (*p* = 0.14) but detected a significant effect for BMI z-score (*p* < 0.001) on CP levels. More precisely, the relationship between CP and BMI z-score was 0.45 without Cobanoglu et al. [12] data (vs. 0.44 with), and 0.42 for boys (vs. 0.39) and 0.47 for girls (vs. 0.48).

### 3.5. Subgroup Analyses

**Figure 2:**
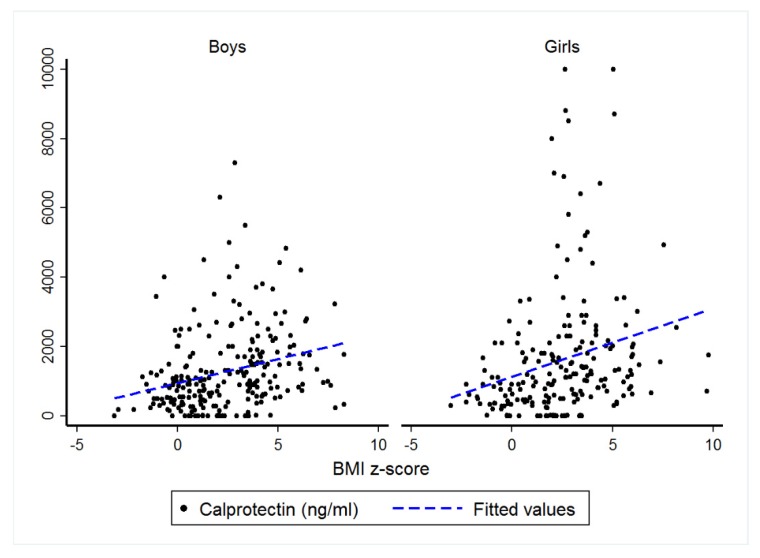
Individual calprotectin values (ng·mL^−1^) according to gender plotted against corresponding BMI z-score in otherwise healthy children.

Multivariate analysis (*n* = 439) by study, age, sex, BMI z-score, and sample type showed no effect for age (*p* = 0.35), sample type (*p* = 0.34), or sex (*p* = 0.054) but detected a significant effect for BMI z-score (*p* < 0.001) on CP levels. Correlation analysis between BMI z-score and corresponding CP values (*n* = 439, Figure 2 and Table 4) revealed significant correlation coefficients of 0.48 among girls (*p* < 0.001) and 0.39 among boys (*p* < 0.001).

Median CP blood levels were similar in children with BMI z-score < 1.34 (581.0 (309.8; 1100.0) ng·mL^−1^) and BMI z-score 1.34–1.65 (703.5 (431.0; 1257.0) ng·mL^−1^; *p* = 0.61). However, they were significantly higher in children with BMI z-score ≥ 1.65 (1301.5 (630.0; 2237.0) ng·mL^−1^) than those with BMI z-score < 1.34 (*p* < 0.001) or BMI z-score 1.34–1.65 (*p* = 0.04).

**Figure 3:**
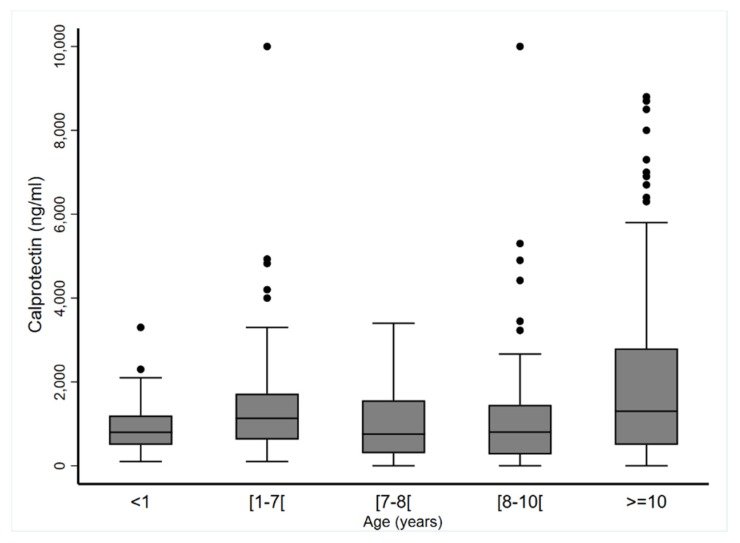
Calprotectin blood concentrations (ng·mL^−1^) according to age (year) grouping (*n* = 593).

To ascertain that age did not affect CP values, we defined five age groups and analyzed the data for girls and boys separately. Figure 3 and Table 5 show that CP blood levels were stable regardless of age (*p* = 0.25 for boys and *p* = 0.97 for girls). In addition, median CP blood levels were similar in plasma (883.9 (468.0; 1535.0) ng·mL^−1^) and in serum (1000.0 (500.0; 1800.0) ng·mL^−1^; *p* = 0.45).

## 4. Discussion

In adults, the strong association between obesity and systemic inflammation has long been recognized and conclusively demonstrated [14,15]. Here, we provide data originating from seven separate studies showing that the link between BMI and subclinical inflammation could be a continuous phenomenon that seems to begin early in life. Furthermore, current findings in the linear increases in CP suggest that even small deviations from ideal body weight may be associated with the initiation of inflammatory processes and thereby foster cardiovascular disease risk [16].

We surmise that in terms of metabolic health it appears as insufficient to consider “obese” and “non-obese” children as a dichotomous condition. Instead, the ideal “wellness state” should therefore include a much more refined evaluation of body composition and adipose tissue distribution along with a combination of several non-overlapping biomarkers that would include CP [17]. Indeed, circulating CP is not only a biomarker of inflammation but also in its protein complex state as a dimer stimulates the recruitment and activation of neutrophils and monocytes. CP is therefore a potent activator of inflammation. In overweight adults, CP correlates with neutrophil blood count and cardiovascular disease, and with other inflammatory molecules such as IL6 or C reactive protein (CRP). It is well known that such proteins are not only surrogate markers but are also directly involved in the pathophysiology of deleterious processes leading to end organ damage [18]. In children, other parameters are also found to linearly correlate with BMI and include blood polymorphonuclear count [19], IL-6, or CRP [20]. Hence, although the correlation coefficients between BMI and CP are not very strong, our data provide further support to our contention that BMI z-score in children may associate with later adverse events even among children whose body weight is within the normative range.

On the other hand, the link between CP levels and BMI z-score shows substantial dispersion, from which we infer that many other factors modulate the intensity of subclinical inflammation. For example, polyunsaturated fatty acids exert different tissue-dependent effects on CP levels [21]. Unlike a previous report [6], we did not find sex to be a significant modulator of CP levels, although a trend was potentially present. We should, however, point out that the effect of gender on CP levels is complicated by the variable dynamic changes associated with transition from pre-pubertal to pubertal states.

From a bioenergetics point of view, one could assume that excessive intake must be metabolized by the body, either toward storage in adipocytes, or toward expenditure primarily via the two major concurrent adaptive systems, i.e., locomotion or physical activity and immunity. This concept of “energetic concurrency” between physical activity and immunity is postulated to account for the reductions in inflammation that occur with exercise. Of note, CP levels will decrease after a six-month physical exercise program in adults with rheumatological conditions [22]. To our knowledge, it is not known whether physical activity levels will inversely correlate with CP levels in otherwise healthy people, especially healthy children.

Our study work has several limitations that are worthy of mention when interpreting the results. Only six studies were available for meta-analysis, and the total number of subjects included was not as extensive as one would prefer. More importantly, the cohort sizes were quite heterogeneous between the different studies. The sample type (serum vs. plasma) and the assay methods used to quantify CP concentrations also diverged in these studies. Pre-analytical processing and assay type may have impacted the levels of CP. This could be the case for the study by Cobanoglu et al. [12] whose CP values were two orders of magnitude lower than other studies. The pre-analytical process in this study used a coagulated sample, which is not the case in other studies. However, the sensitivity analysis without data of Cobanoglu et al. [12] showed no difference with reported results. In addition, only published studies in English were considered, and we could not address confounding factors. Therefore, significant and important data obtained in either unpublished studies or in studies written in other languages could have been omitted.

## 5. Conclusions

Our study shows that in healthy children, the blood levels of CP seem to be correlated with the BMI z-score, suggesting that sub-clinical inflammation is a continuous process influenced by adiposity even when the criteria for overweight or obesity are not met, and that such changes are detectable even in young children. Our findings therefore place into question the relevance of defining a rigid threshold to define overweight or obese, and rather propose that we consider BMI z-score as a continuous variable when estimating the risk of subsequent health impairments.

## Figures and Tables

**Table 1:** Characteristics of included studies.

Study/Year	Population and Condition	Age	Matrix	Test Kit	Sample Condition and Storage	Lower Detection Limit	Calprotectin Values
Kim et al. 2010 [9]	Mod./sev. OSA (*n* = 34); Mild OSA (*n* = 106); Controls (*n* = 115)	Mod./sev. OSA: 7.20 ± 1.96 yr.; Mild OSA: 7.65 ± 1.55 yr.; Controls: 7.81 ± 1.44 yr.	Plasma	ELISA (R&D Systems, Minneapolis, USA)	Fasting blood sample centrifuged and frozen at −80 °C	0.4 µg·mL^−1^	Mean ± SD in µg·mL^−1^ Mod./sev. OSA: 1.73 ± 0.92; Mild OSA: 1.27 ± 0.87; Controls: 1.02 ± 0.85
Kim et al. 2010 [10]	Mod./sev. OSA (*n* = 26); Mild OSA (*n* = 85); Controls (*n* = 102)	Mod./sev. OSA: 7.19 ± 1.83 yr.; Mild OSA: 7.79 ± 1.57 yr.; Controls: 7.71 ± 1.29 yr.	Plasma	ELISA (ALPCO Diagnostics, Salem, USA)	Fasting blood sample centrifuged and frozen at −80 °C	0.4 µg·mL^−1^	Mean ± SD in µg·mL^−1^ Mod./sev. OSA: 1.82 ± 0.97; Mild OSA: 1.28 ± 0.91; Controls: 1.00 ± 0.84
Terrin et al. 2011 [11]	Low body weight newborns: Septic group (*n* = 62); Non septic group (*n* = 29); Controls (*n* = 110)	Septic group: 8.7 d (95% CI: 7.2–10.1); Non septic group: 6.8 d (95% CI: 5.5–8.0); Controls: 6.9 d (95% CI: 6.1–7.7)	Serum	ELISA (Calprest, Eurospital, Trieste, Italy)	Collected in EDTA tube, centrifuged for 10 min at 10,000 rpm, stored at −20 °C	1.6 ng·mL^−1^	Mean (95% CI) in µg·mL^−1^ Septic: 3.1 (2.9–3.4); Non septic: 1.1 (0.9–1.2); Controls: 0.91 (0.8–1.0)
Cobanoglu et al. 2012 [12]	Group 1 (*n* = 51): not exposed Group 2 (*n* = 46): exposed to second-hand smoke at home	Group 1: 8.6 ± 1.6 yr.; Group 2: 8.4 ± 1.3 yr.	Serum	ELISA (Cusabio Biotech, China)	Coagulate for 30 min, centrifuged for 15 min at 10,000 rpm, frozen at −20 °C	2 ng·mL^−1^	Median (min–max) in ng·mL^−1^ Group 1: 0.00 (0.00–17.82); Group 2: 2.25 (0.00–106.87)
Schellekens et al. 2013 [13]	Acute abdominal complaints suspected for appendicitis (*n* = 233); Controls (*n* =52)	33 yr. (min–max: 5–79)	Plasma	ELISA (Hbt, Uden, the Netherlands)	Collected in EDTA tube, centrifuged for 12 min at 2100 rpm, frozen at −20 °C	10 ng·mL^−1^	Median (IQR) in ng·mL^−1^ AA: 320.9 (193.4–492.4); Controls: 219.9 (104.3–323.0)
Calcaterra et al. 2018 [6]	Normal weight (*n* = 39); Overweight (*n* = 36); Obesity (*n* = 56)	NW: 12.3 ± 5.1 yr.; OW: 11.4 ± 4.0 yr.; Ob: 11.4 ± 3.0 yr.	Serum	ELISA (Calprest, Eurospital, Trieste, Italy)	ND	0.3 µg·mL^−1^	Median (IQR) in µg·mL^−1^ NW: 1.1 (0.9–2.1); OW: 1.85 (1.1–4.7); Ob: 2.65 (1.6–4.2)

Mod./Sev.: moderate to severe; yr.: years; d: days; CI: confidence interval; IQR: interquartile range; SD: standard deviation; OSA: obstructive sleep apnea; AA: acute appendicitis; NW: normal weight; OW: overweight; Ob: Obesity; ND: not done; com.: commercially available.

**Table 2:** Patient characteristics based on available data.

Study	Year	Country	Sample Type	Number of Participants	Girls/Boys	CP Values in Blood (ng·mL^−1^)	Age (Years)	BMI z-Score
Kim J et al. ^‡^ [9,10]	2010	United States	Plasma	252	106/146	1194.7 ± 856.3	7.6 ± 1.5	2.82 ± 2.59
Terrin G et al. [11]	2011	Italy	Serum	139	58/81	952.5 ± 543.5	0.018 ± 0.010	NK
Cobanoglu et al. [12]	2012	Cyprus	Serum	51	25/26	1.72 ± 3.39	8.4 ± 1.3	1.82 ± 1.56
Schellekens D et al. [13]	2013	Netherlands	Plasma	4	0/4	193.6 ± 97.4	15.0 ± 2.6	NK
Calcaterra et al. [6]	2018	Italy	Serum	122	65/57	2680.3 ± 2169.8	11.1 ± 3.2	2.36 ± 1.69
Merlin et al. ^‡‡^	2018	France	Plasma	26	16/10	419.9 ± 264.5	10.7 ± 3.3	0.09 ± 1.47

^‡^ Data from Kim et al. [9,10] came from 2 studies and unpublished results; no distinction was done between results from different sources. ^‡‡^ unpublished data from our center. Results are presented as mean ± SD. CP: calprotectin; NK: not known.

**Table 3:** Mean calprotectin values for all studies.

Authors	*n*	Mean	SD	Q1	MED	Q3	P5	P95	MIN	MAX	CV
Kim et al. [9,10]	252	1194.73	856.63	550.00	963.50	1566.50	250.00	2789.00	100.00	4930.00	0.72
Terrin et al. [11]	139	952.52	543.52	500.00	800.00	1200.00	200.00	2000.00	100.00	3300.00	0.57
Cobanoglu et al. [12]	51	1.73	3.40	0.00	0.00	1.90	0.00	6.80	0.00	17.80	1.97
Schellekens et al. [13]	3	193.63	97.41	113.10	165.90	301.90	113.10	301.90	113.10	301.90	0.50
Calcaterra et al. [6]	122	2680.33	2169.88	1100.00	1950.00	3400.00	600.00	7300.00	400.00	10,000.00	0.81
Merlin et al.	26	419.91	264.47	303.30	336.80	460.70	177.30	883.90	140.80	1415.60	0.63
Meta-Analysis	593	880.75	255.36	482	900	1700					

Intraclass correlation coefficient (ICC): 36%. SD: standard deviation; Q1: first quartile; MED: median; Q3: third quartile; P5: fifth percentile; P95: ninety-fifth percentile; Min: minimum; MAX: maximum; CV: coefficient of variation.

**Table 4:** Regression table for univariate and multivariate results.

	Univariate Analysis ^1^ Correlation Coefficient	Multivariable Analysis ^2^ Coefficient Correlation
Boys	0.39	0.118 (0.076; 0.159), *p* < 0.001
Girls	0.48	0.116 (0.072; 0.160), *p* < 0.001

^1^ Spearman correlation coefficient; ^2^ Linear mixed models with CP log-transformed and adjusted on age and sample type in addition to study as random-effect.

**Table 5:** Calprotectin blood concentrations (ng·mL^−1^) according to sex and age groups.

Year(s)	<1	(≥1–<7)	(≥7–<8)	(≥8–<10)	≥10	*p*
Boys						
*n*	81	68	48	64	62	0.25
Mean ± SD	849.4 ± 455.3	1356.7 ± 949.8	802.9 ± 850.5	977.2 ± 937.3	1752.8 ± 1666.1	
Median (min–max)	800 (100–2300)	1165 (100–4824)	498.8 (0–2789)	812 (0–4420)	1150 (0–7300)	
Girls						
*n*	58	48	43	52	69	0.97
Mean ± SD	1096.5 ± 622.9	1395.3 ± 1556.0	1169.1 ± 936.5	1216.7 ± 1658.5	2277.1 ± 2400.3	
Median (min–max)	1100 (100–3300)	1060 (189–10,000)	978 (0–3400)	803.5 (0–10,000)	1400 (0–8800)	

*p*-Values were estimated using linear mixed models with CP log-transformed and study as random-effect.

## Supplementary Materials

The following are available online at https://www.mdpi.com/2077-0383/9/3/857/s1, Figure S1: The Newcastle–Ottawa Scale (NOS) for assessing the quality of studies.

## Abbreviations

BMI	body mass index
CP	calprotectin
CRP	C-reactive protein
ICC	intraclass correlation coefficient
ELISA	enzyme linked immunosorbent assay
MRP8/14	myeloid related protein 8/14
NOS	Newcastle–Ottawa scale
PRISMA	Preferred Reporting Items for Systematic Reviews and Meta-Analyses
SD	standard deviation
TLR-4	toll-like receptor 4
