# Supplementary material for: Body Mass Index and Calprotectin Blood Level Correlation in Healthy Children: An Individual Patient Data Meta-Analysis
Source: J Clin Med. 2020 Mar 20;9(3):857. doi: 10.3390/jcm9030857 (PMC7141538; doi:10.3390/jcm9030857)
Supplement: Supplementary file 1 [file jcm-09-00857-s001.pdf]

Supplementary file

**Supplementary table S1**: The Newcastle-Ottawa Scale (NOS) for assessing the quality of studies.				
	**Selection**	**Comparability**	**Exposure**	**Total quality score**
Kim et al. 2010 [9]	***	**	**	7
Kim et al. 2010 [10]	***	**	**	7
Terrin et al. 2011 [11]	***		****	7
Cobanoglu et al. 2012 [13]	***		****	8
Schellekens et al. 2013 [12]	****	**	***	9
Calcaterra et al.2018 [6]	***		****	7

The PubMed strategy is described below. We used the following search terms and strategies for serum calprotectin and plasma calprotectin respectively: ("serum"[MH] OR "blood serum"[TW] OR "sera"[TW] OR "serum, blood"[TW] OR "serums"[TW] OR "serum"[TW]) AND (“calprotectin”[TW] OR “calprotectine”[TW] OR “calprotectins”[TW] OR “Leukocyte L1 Antigen Complex”[TW] OR “Leukocyte L1 Antigen Complex”[MH] OR “MRP8/14”[TW] OR “MRP8-14”[TW] OR “myeloid-related protein-8/14”[TW] OR “calgranulin A”[TW] OR “calgranulin B”[TW]) AND ("child"[MH] OR "progeny"[TW] OR "minor"[TW] OR "child"[TW] OR "childs"[TW] OR "children"[TW] OR child, “preschool”[TW] OR "adolescent"[MH] OR "male adolescent"[TW] OR "teenager"[TW] OR "male adolescents"[TW] OR "female adolescent"[TW] OR "adolescents"[TW] OR "youths"[TW] OR "adolescent, male"[TW] OR "teen"[TW] OR "teenagers"[TW] OR "female adolescents"[TW] OR "adolescent, female"[TW] OR "adolescent"[TW] OR "infant"[MH] OR "infants"[TW] OR "baby"[TW] OR "infant"[TW] OR "infants, newborn"[TW] OR "newborn infant"[TW] OR "newborn infants"[TW] OR "neonate"[TW] OR "neonates"[TW] OR "newborn"[TW] OR "infant, newborn"[TW] OR "newborns"[TW]) and ("plasma"[MH] OR "blood plasma"[TW] OR "fresh frozen plasmas"[TW] OR "plasmas, fresh frozen"[TW] OR "plasma, fresh frozen"[TW] OR "fresh frozen plasma"[TW] OR "plasma"[TW] OR "plasmas, blood"[TW] OR "plasma, blood"[TW] OR "frozen plasma, fresh"[TW] OR "frozen plasmas, fresh"[TW] OR

"blood plasmas"[TW] OR "plasmas"[TW]) AND (“calprotectin”[TW] OR “calprotectine”[TW] OR “calprotectins”[TW] OR “Leukocyte L1 Antigen Complex”[TW] OR “Leukocyte L1 Antigen Complex”[MH] OR “MRP8/14”[TW] OR “MRP8-14”[TW]) AND ("child"[MH] OR "progeny"[TW] OR "minor"[TW] OR "child"[TW] OR "childs"[TW] OR "children"[TW] OR child, preschool[TW] OR "adolescent"[MH] OR "male adolescent"[TW] OR "teenager"[TW] OR "male adolescents"[TW] OR "female adolescent"[TW] OR "adolescents"[TW] OR "youths"[TW] OR "adolescent, male"[TW] OR "teen"[TW] OR "teenagers"[TW] OR "female adolescents"[TW] OR "adolescent, female"[TW] OR "adolescent"[TW] OR "infant"[MH] OR "infants"[TW] OR "baby"[TW] OR "infant"[TW] OR "infants, newborn"[TW] OR "newborn infant"[TW] OR "newborn infants"[TW] OR "neonate"[TW] OR "neonates"[TW] OR "newborn"[TW] OR "infant, newborn"[TW] OR "newborns"[TW]).
